# The Diagnostic Yield of Cone Beam CT Combined With Radial-Endobronchial Ultrasound for the Diagnosis of Peripheral Pulmonary Nodules: Systematic Review and Meta-Analysis

**DOI:** 10.1016/j.chpulm.2024.100037

**Published:** 2024-01-24

**Authors:** Michael V. Brown, Arash Badiei, Matthew Arnold, Hubertus Jersmann, Thomas Sullivan, David Fielding, Phan Nguyen

**Affiliations:** a Department of Thoracic Medicine, Royal Adelaide Hospital, Adelaide, SA; b Faculty of Health and Medical Sciences, Adelaide Medical School, University of Adelaide, Adelaide, SA; c School of Public Health, Faculty of Health and Medical Sciences, University of Adelaide, Adelaide, SA; d Department of Thoracic Medicine, Royal Brisbane and Women’s Hospital, Brisbane, QLD, Australia

**Keywords:** cone beam CT scan, lung cancer, peripheral pulmonary nodule, radial endobronchial ultrasound, solitary pulmonary nodule

## Abstract

**Background:**

Identification of peripheral pulmonary nodules (PPNs) is becoming increasingly common with modern imaging and lung cancer screening programs. Navigational bronchoscopy has been developed to augment the diagnostic yield of sampling these nodules. Cone beam CT (CBCT) scan is one navigational tool which can be used alongside the historical criterion standard of fluoroscopy and radial endobronchial ultrasound (r-EBUS).

**Research Question:**

What is the diagnostic yield and safety profile of combining CBCT scan with r-EBUS for the diagnosis of PPNs?

**Study Design and Methods:**

Embase, PubMed, and Cochrane Central Register of Controlled Trials were searched in March 2023. Eligible studies used CBCT scan with r-EBUS as the primary navigation technique. The primary outcome, diagnostic yield, was analyzed using random effects meta-analysis. Additional subgroup analysis was based on the use of additional navigational technologies. Risk of bias was assessed using the Critical Appraisal Skills Programme tool for diagnostic studies. The Grading of Recommendations Assessment, Development, and Evaluation tool was used to assess the quality of outcomes.

**Results:**

Fourteen studies (865 patients and 882 lesions) were included. The risk of bias was significant as assessed using the Critical Appraisal Skills Programme tool, which identified multiple confounders. The pooled diagnostic yield of combined CBCT scan and r-EBUS-guided biopsy for the diagnosis of PPNs was 80% (95% CI, 76%-84%). Subgroup analysis of diagnostic yield for CBCT scan and r-EBUS alone was 80% (95% CI, 76%-83%). The diagnostic yield of CBCT scan and r-EBUS combined with additional navigational technology (electromagnetic navigational bronchoscopy, virtual bronchoscopic navigation, and robotic-assisted bronchoscopy) was 80% (95% CI, 73%-87%). The quality of outcomes was assessed as low to very low using the Grading of Recommendations Assessment, Development, and Evaluation tool. There was a 2.01% pneumothorax rate and 1.08% bleeding rate. Although heterogeneously reported, the total radiation dose was between 19.59 and 85.9 Gy.cm^2^, resulting in an approximate effective dose range of 3.1 to 13.8 mSv.

**Interpretation:**

CBCT scan and r-EBUS for the diagnosis of PPNs has a high diagnostic yield and acceptable safety profile. Studies showed moderate heterogeneity with significant bias; hence, generalizability of the study is limited and further prospective trials are required.

**Clinical Trial Registration:**

PROSPERO; No.: CRD42023410221; URL: https://www.crd.york.ac.uk/prospero/.

Peripheral pulmonary nodules (PPNs) are focal parenchymal opacities typically identified on chest imaging. By definition, a PPN is a < 3 cm opacity completely surrounded by pulmonary parenchyma and cannot be visualized endobronchially during bronchoscopic examination.^1^ The prevalence of pulmonary nodules is estimated to be 14% to 36%. This is likely an underestimation, and the prevalence is expected to increase with the advent of improved imaging technology and adoption and implementation of lung cancer screening programs globally.^2^ The investigation of a PPN can be challenging. A biopsy of a nodule is indicated when a patient has risk factors, or the nodule has characteristics, that increase the probability of malignancy.^1^

Transbronchial biopsy under fluoroscopic guidance has been an accepted method for diagnosing PPNs since the early 1970s.^3^ Fluoroscopy provides a real-time 2-D radiograph-based assessment of the biopsy tool in relation to the target PPN. However, fluoroscopy comes with the known limitations of 2-D radiograph-based imaging, with some PPNs not being apparent at the time of imaging, and there being no direct way of confirming that the biopsy tool is within the 3-D nodule.^4^ Radial endobronchial ultrasound (r-EBUS) guide sheath biopsy combined with fluoroscopic guidance results in superior diagnostic yield compared with conventional transbronchial biopsies.^5^ This approach allows localization of the PPN using ultrasound and placement of a guide sheath for subsequent biopsies. However, the same inherent limitations of 2-D radiograph imaging remain with the fluoroscopic guidance used with this approach. This is likely a reason that the diagnostic yield of r-EBUS with fluoroscopy guidance appears to have plateaued at approximately 70%.^6^ The alternative method for the diagnosis of PPNs is CT-guided transthoracic needle aspirate. This technique has a diagnostic accuracy of 92.1%; however, it comes at the expense of morbidity with a pneumothorax rate of 20.5% to 25.9%^7^ in comparison with a pneumothorax rate for fluoroscopically guided r-EBUS of approximately 1%.^8^

To improve on the bronchoscopic diagnostic yield, novel navigational techniques have been developed. Bronchoscopic navigational techniques refer to tools that facilitate one or more of navigation (ability to reach a target), confirmation (confirmation that sampling tools are in contact with the target), and acquisition (ability to obtain diagnostic samples).^9^ A number of navigational technologies now exist. Electromagnetic navigation bronchoscopy (ENB) reportedly has a diagnostic yield of 65% to 70%.^10^^,^^11^ There are limited data for the combination of r-EBUS and ENB; however, a few studies have reported a diagnostic yield up to 88%.^12^ Virtual bronchoscopy/virtual bronchoscopic navigation (VBN) and robotic-assisted bronchoscopy (RAB) are alternative navigational modalities.^13^^,^^14^ Other adjuncts have also been used alone or in combination with these navigational techniques. Augmented fluoroscopy (AF) fuses the preoperative CT scan and intraoperative fluoroscopy to provide an enhanced overlapping image on the 2-D fluoroscopy that facilitates guidance and localization of the nodule.^15^ Ultrathin bronchoscopy (UTB) and novel sampling tools (eg, transbronchial access tools, transbronchial needle aspiration, transbronchial cryobiopsy^16^, ^17^, ^18^) also appear to have a role. Postbiopsy, rapid onsite cytologic examination (ROSE) may further increase the diagnostic yield.^19^

Recently, 3-D cone beam CT (CBCT) scan has been used for navigation. CBCT navigation involves a compact CT system within a C-arm or O-arm that provides cross-sectional, real-time evidence of the bronchoscope and biopsy tools location relative to a nodule. Cone beam differs from traditional CT scan in that it emits a cone-shaped x-ray beam that acquires images over a large volume in a single scan. Traditional CT scan emits a fan-shaped x-ray beam and moves around the patient acquiring a single axial image slice per scan. The individual axial slices are then reconstructed to create the larger volume image. Traditional CT scan provides greater image resolution; however, this comes at the expense of significantly more radiation.^20^ CBCT scan has been used both in combination with r-EBUS and independently alongside multiple other navigation techniques.^18^^,^^21^, ^22^, ^23^, ^24^ The intent has been to improve on the standard 2-D fluoroscopy-guided r-EBUS by providing real-time evidence of biopsy tools in the lesion.^21^^,^^25^ CBCT scan is defined as a navigational tool because of its role in stimulating renavigation based on confirmation of the biopsy tools positioning relative to the lesion. Additionally, it can be used as a platform for AF to delineate and overlay the lesion and pathway toward the lesion. The purpose of this systematic review and meta-analysis is to assess the diagnostic yield of combining CBCT scan with r-EBUS. To our knowledge, this is the first time this combination of technologies has undergone systematic review and meta-analysis.

## Study Design and Methods

### Protocol and Registration

We developed a review protocol in line with the Preferred Reporting Items for Systematic Reviews and Meta-Analyses statement prior to commencement of the systematic review. This protocol is registered on the PROSPERO database (registration No. CRD42023410221).

### Eligibility Criteria

English-language studies published between 1990 and the date of search (March 22, 2023) and those involving adults aged > 18 years were considered eligible. Studies were required to have sufficient data for calculating the diagnostic yield when both CBCT scan and r-EBUS were used for the biopsy of PPNs. Studies that included additional navigational modalities (eg, ENB, RAB, VBN) were included. Similarly, studies that included other diagnostic adjuncts (eg, AF, ROSE, UTB) were also eligible. Trial designs included randomized controlled trials, cohort studies, case-control studies, and case series.

We excluded studies that did not report the utilization of CBCT scan or r-EBUS on humans and studies that did not satisfy the definition of a pulmonary nodule (mean or median nodule size < 30 mm). We excluded studies in which diagnostic yield was not reported or could not be calculated. To avoid errors in translation and geographic variation in practice, studies not in English were also excluded. Literature not subject to peer review, review articles, editorials, and abstracts without any case description were ineligible. Studies with fewer than five patients were excluded.

### Information Sources and Search Strategy

**Figure 1:**
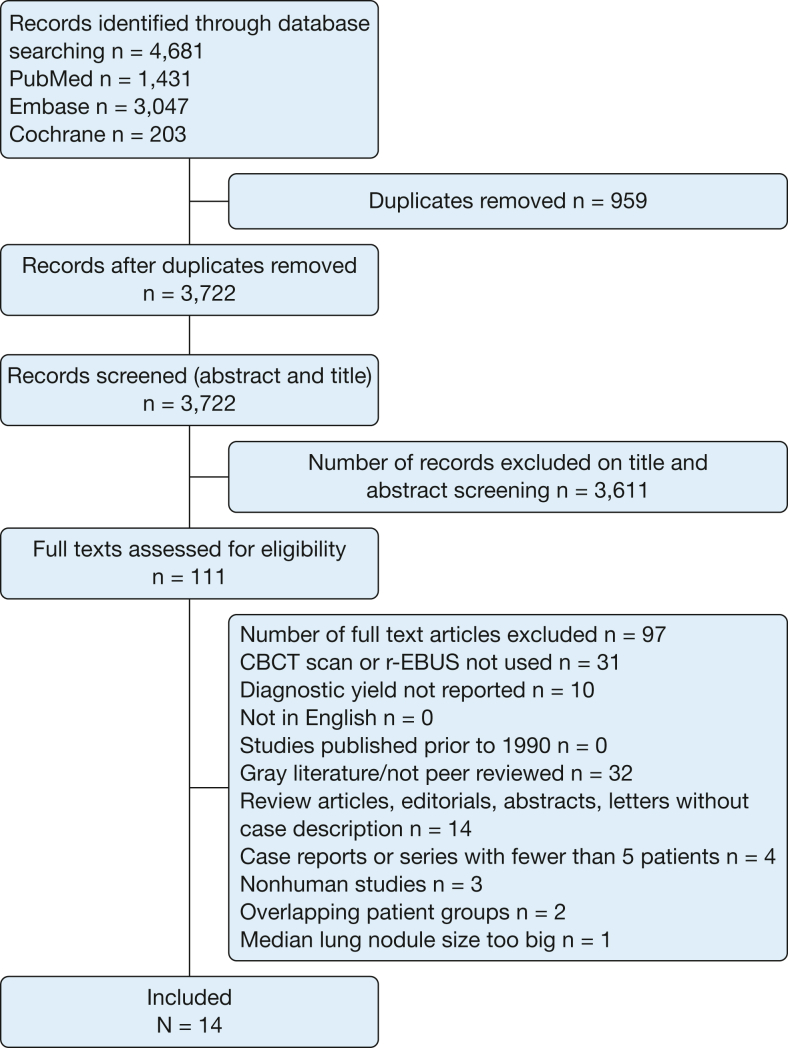
Systematic review method. CBCT = cone beam CT; r-EBUS = radial endobronchial ultrasound.

Using the search strategies outlined in e-Table 1, we searched Embase, PubMed, and Cochrane Central Register of Controlled Trials to identify original, peer-reviewed, full-length, human patient articles describing the use of CBCT scan with r-EBUS for the diagnosis of PPNs. This strategy was used across the three databases on March 22, 2023, to yield the articles screened for inclusion in the review (Fig 1).

### Selection Process

Search results from each database were imported into EndNote 20 (Clarivate Analytics). Key word searching was also performed to identify new studies that had not yet been assigned indexing terms for the databases. Reference lists from key articles were analyzed to identify further studies that may have been relevant to the review. Titles and abstracts were reviewed by one author (M. V. B.), who was not anonymized to journal articles, authors, or institutions. Articles selected for full text review were reviewed by two authors (M. V. B. and M. A.). Any disagreement between the two authors was resolved by a consulting third party (P. N.). The list of references was reviewed by all authors to ensure consensus was reached.

### Data Collection Process

For every article, the Cochrane data extraction template for included studies was used for data collection. Data were extracted by one reviewer (M. V. B.) and confirmed by a second reviewer (M. A.). Clarification or disagreement of any data point was resolved with discussion with a third reviewer (P. N.).

### Data Items

For every included study, author, year, and type of data collection (retrospective vs prospective) were collated. Demographic data on the number of patients, sex, and age of patients were extracted (Table 1). A description of the navigation technique, the role of the CBCT scan, the sampling tools used for biopsy, and the use of diagnostic adjuncts were also collected (Table 2). The diagnostic accuracy reported in this paper as a percentage and absolute number was gathered as the primary end point. Where reported, additional information about the characteristics of the sampled pulmonary nodules was also included (eg, size of the nodule, distance from the pleura, location, percentage of solid nodules, presence of bronchus sign, fluoroscopic visibility), in addition to data on complications, number of CBCT scans, radiation dose, and bronchoscopy duration (Table 1).

**Table 1:** Data Extraction: Study Characteristics

Study	Arms	No. of Patients Included	No. of Male Patients	Age, y	Size of PPN, mm	Distance From Pleura, mm	Nodule located in the Upper Lobe (%)	Radiation Dose	No. of CBCT Scans	Bronchoscopy Duration, min	No. of Solid Lesions (%)	No. With Bronchus Sign Positive (%)	Fluoroscopically Visible	Complications
Casal et al^26^	CBCT scan + r-EBUS	20	5	Median, 70 (range, 48-86)	Median, 21 (range, 11-30)	Median, 21 (range, 0-28)	12 (60)	CBCT scan: 50.45 Gy.cm^2^ (range, 5.43-66.75); total exposure: total P_KA_: 64.57 Gy.cm^2^ (range, 6.14-66.75)	Mean, 1.5 (range, 1-2)	Total median time, 62.5 (range, 49-96)	13 (65)	12 (60)	8/20 (40)	1 pneumothorax
Salahuddin et al^27^	CBCT + r-EBUS	51	25	N/A	Mean ± SD 26 +/-13	Mean ± SD 15 ± 14	38 (74)	DAP from CBCT spins: 11.35 Gy.cm^2^; total exposure: mean± SD 41.92± 26.19 Gy.cm^2^ (range, 9.1-113.08)	Mean ± SD, 1.82 ± 1.01 Median 1 (range, 1-5)	Total median time, 85 (range, 24-144)	37 (72.5)	39 (75)	N/A	1 pneumothorax
Yu et al^34^	CBCT scan + AF + r-EBUS	53	30	Mean, 64.6 (range, 31-93)	Median, 28 (range, 1-6.9)	N/A	29 (54.8)	CBCT: P_KA_ mean ± SD, 16.4 ± 8.01 Gy.cm^2^ (range, 3.63-39.82); total exposure ± SD: 19.59 ± 11.68 Gy.cm^2^ (range, 4.14-64.13)	Mean, 1 (range, 1-2)	Total mean bronchoscopy time ± SD, 20.4 ± 8; total mean examination time ± SD, 40.8 ± 15.0	46 (86.8)	40 (75.5)	29 (54.7)	2 bleeding
	r-EBUS	53	34	Mean, 63.5 (range, 29-88)	Median 29 (range, 1.0-6.9)	N/A	29 (54.7)	Not applicable	Not applicable	Total mean bronchoscopy time ± SD, 24.1 ± 9.6; total examination time ± SD, 41.3 ± 13.3	50 (94.3)	36 (67.9)	N/A	2 bleeding, 1 pneumothorax
Lin et al^33^	CBCT -AF + r-EBUS	115	50	Mean, 65.1 (range, 28-91)	Mean, 24 (range, 6.0-62.0)	N/A	64 (55.6)	CBCT scan: 22.78 Gy.cm^2^ (range, 7.26-119.16); total exposure: 25.48 Gy.cm^2^ (range, 9.46-123.41)	mean 1 (19/115 had intraprocedural CBCT scan)	Total mean procedure time, 41.9 (range, 16-109)	88 (76.5)	95 (82.6)	80 (69.6)	5 bleeding, 0 pneumothorax, 4 fever
	r-EBUS	121	68	Mean, 67.0 (range, 31-94)	Mean, 30.6; (range, 6.2-64.2)	N/A	53 (43.8)	Not applicable	Not applicable	Total mean procedure time, 34.4 (range, 12-78)	105 (86.9)	108 (89.3)	90 (74.4)	3 bleeding, 3 pneumothorax, 8 fever
Dibardino et al^35^	CBCT scan + r-EBUS	22	10	Age 25-61: 30, Age 62-68: 31, Age 69-73: 28, Age 74-89: 27	Median, 19.5 mm (IQR, 15-27.5)	N/A	7 (25.9)	Total exposure: median, 70.42 Gy.cm^2^ (IQR, 42.49-99.70); median, 11.97 mSv (IQR, 7.22-16.95)	Median, 2 (range, 1-4)	N/A	N/A	19 (70.4)	14 (51.9)	1 pneumothorax
	CBCT scan + r-EBUS + VBN	5												
	CBCT scan + UTB + r-EBUS	26	16			N/A	19 (63.3)			N/A	N/A	21 (70)	20 (66.6)	5 pneumothorax
	CBCT + UTB + r-EBUS + VBN	4												
	r-EBUS	32	29			N/A	33 (55.9)	…	…	N/A	N/A	47 (79.7)	38 (64.4)	3 pneumothorax, 1 pneumomediastinum
	r-EBUS + VBN	27												
Kawakita et al^36^	VBN + UTB + r-EBUS + CBCT scan	20	14	Mean, 70.7 (range, 44-86)	Mean, 24.8 (range, 10-46)	N/A	10 (50)	N/A	Mean, 1.3 (range, 1-3)	Total mean procedure time, 44 (range, 32-93)	16 (80)	19 (95)	11 (55)	1 pneumothorax
Katsis et al^37^	ENB + CBCT scan + r-EBUS	26	17	Mean, 64 (range, 31-81)	Mean, 12.8 (range, 9-16.6)	Mean ± SD, 12 ± 14	18 (69.2)	Total exposure ± SD 259.6 ± 208.2 mGy	N/A	Total mean procedure time ± SD, 78.7 ± 28.1 (includes mediastinal staging/additional diagnostic procedures in 62.5%)	N/A	5 (17)	N/A	0
Kheir et al^38^	ENB + CBCT scan + r-EBUS	31	19	Mean ± SD, 67.7 ± 8.2	Median, 16 (IQR, 12.6-25.5)	Median, 8 (IQR, 0-20)	21 (67.7)	N/A	Mean, 1.3	Total median procedure time, 74 (IQR, 61-87)	19 (61.3)	14 (45.2)	8 (25.8)	2 pneumothorax
	ENB + r-EBUS	31	28	Mean ± SD, 64.5 ± 7.3	Median, 21.5 (IQR, 16-27)	Median, 19 (IQR, 0-28)	20 (64.%)	Not applicable	Not applicable	Total median procedure time, 90 (IQR, 72-119)	18 (58.1)	13 (41.9)	13 (41.9)	2 pneumothorax
Podder et al^39^	ENB + CBCT scan + r-EBUS	14	8	Mean, 71 (range, 62-84)	Mean ± SD, 21.7 ± 14.9 × 13.2 ± 4.8 (axial × coronal)	N/A	11 (65)	Total exposure ± SD, 858.5 ± 553 mGy	Mean ± SD, 3.5 ± 1.5	N/A	14/17 (82.4)	11/17 (64.7)	N/A	0
Sobieszczyk et al^40^	ENB + CBCT scan + r-EBUS	22	8	Mean, 69 (range, 55-83)	Mean, 21 (range, 0.7-5.2)	N/A	15 (68.2)	N/A	Mean ± SD, 2.63 ± 1.06 (range, 1-4)	Total mean procedure time ± SD, 79.95 ± 21.15 (range, 50-124)	N/A	N/A	12 (55)	0
Verhoeven et al^41^	Primary CBCT scan group	150	114	Mean, 65 (range, 36-85)	Median, 13 (range, 5-65)	N/A	152 (61.3)	Total DAP ± SD: 25.4 ± 5.8 mSv to 47.5 ± 14.3 mSv; depending on the trial period	Mean ranges from 2.0 to 2.93 scans depending on the trial period	Mean navigation time, 29 (range, 4-100); mean biopsy time ± SD, 25.6 ± 10.74	N/A	60.90%	N/A	N/A
	Primary ENB group	58												
Cumbo-Nacheli et al^42^	RAB + CBCT scan + r-EBUS	20	7	Mean ± SD, 70 ± 7	Mean ± SD, 22 ± 7	N/A	16 (80)	N/A	Mean, 2.4	Mean navigation time, 9.8 (range, 3-41); mean procedure time, 36.4 (range, 15-66)	17 (85)	10 (50)	N/A	0
Styrvoky et al^43^	SSRAB + CBCT scan + r-EBUS	198	94	Mean ± SD, 67.1 ± 12.9	Mean ± SD, 22.6 ± 13.3 (range, 7-73)	N/A	126/209 (60.3)	N/A	N/A	N/A	154/209 (73.7)	126 (60.3)	N/A	2 pneumothorax
Reisenauer et al^44^	SSRAB + CBCT scan + r-EBUS	30	17	Mean, 69.3 (range, 35-87)	Median, 17.5 (range, 10-30)	Mean ± SD, 14.9 ± 11.8	18 (60)	Total exposure ± SD, 50.3 ± 32.0 Gy.cm^2^	Mean ± SD, 2.5 ± 1.6	Total mean procedure time ± SD, 55.4 ± 35.1	23 (76.7)	12 (40)	N/A	2 had hemodynamic instability and procedures were not performed

AF = augmented fluoroscopy; CBCT = Cone beam CT; DAP = dose area product; ENB = electromagnetic navigation bronchoscopy; IQR = interquartile range; N/A = not available; P_KA_ = kerma area product; PPN = peripheral pulmonary nodule; r-EBUS = radial endobronchial ultrasound; RAB = robotic-assisted bronchoscopy; SSRAB = shape sensing robotic-assisted bronchoscopy; UTB = ultrathin bronchoscope; VBN = virtual bronchoscopic navigation.

**Table 2:** Data Extraction: Study Design and Description of Navigation, Sampling, and Diagnostic Adjuncts

Study	Design	Arms	Navigation Description	Role of CBCT Scan	Sampling Tools	Diagnostic Adjuncts
Casal et al^31^	Pr	CBCT scan + r-EBUS	Flexible bronchoscopy (Olympus BF-P190; Olympus America Inc), r-EBUS probe (UM-S20-17S; Olympus America Inc), fluoroscopy	CBCT scan (Siemens Artis dTA angiography system; Siemens Healthineers) to confirm navigation success. If navigation unsuccessful, then renavigation with ultrathin bronchoscope BF-XP-190 (Olympus America Inc). Repeat CBCT scan as needed.	TBNA, forceps, brushing	ROSE
Salahuddin et al^32^	Re	CBCT scan + r-EBUS	Flexible bronchoscope (BF-P190F; Olympus; or BF-MP190F; Olympus), r-EBUS (UM-S20-17S; Olympus), fluoroscopy	m-CBCT scan (Cios Spin; Siemens Healthineers) to confirm navigation success.	TBNA (21G PeriView, FLEX needle; Olympus), forceps, brushing	ROSE
Yu et al^34^	Re	CBCT scan + AF + r-EBUS	CBCT scan (Artis Zee; Siemens)-augmented fluoroscopy (Syngo X-workplace; Siemens) and then flexible bronchoscope (BF-P260F or BF-290Q; Olympus) + r-EBUS (UM-S20-17S; Olympus)	Rotation of the C-arm to confirm tool in lesion.	Forceps, brushings	Nil
		r-EBUS	Flexible bronchoscope (BF-P260F or BF-290Q; Olympus), r-EBUS (UM-S20-17S; Olympus)	Nil		
Lin et al^33^	Re	CBCT-AF + r-EBUS	CBCT (Artis Zee Ceiling; Siemens)-AF (Syngo iGuide Toolbox; Siemens), bronchoscope (BF-Q290 or BF-P290; Olympus), r-EBUS (UM-S20-17S; Olympus)	19/115 had intraprocedural CBCT scan.	Forceps	ROSE
		r-EBUS	Bronchoscope (BF-Q290 or BF-P290; Olympus), r-EBUS (UM-S20-17S; Olympus)	Nil		
Dibardino et al^35^	Re	CBCT scan + r-EBUS	Flexible bronchoscope (BF-1TH190 or BF-P190; Olympus), r-EBUS (20-MHz 17S; Olympus) (± guide sheath)	CBCT (Artis Zeego; Siemens)-AF (Syngo X-workplace; Siemens). Repeat CBCT scan as required for tool in lesion.	TBNA (21G PeriView FLEX needle; Olympus), brushing, forceps, BAL	Nil
		CBCT scan + r-EBUS + VBN	VBN navigation added to previously mentioned technique			
		CBCT scan + UTB + r-EBUS	Ultrathin flexible bronchoscope (BF-MP190F; Olympus), r-EBUS (± guide sheath)	CBCT (Artis Zeego; Siemens)-AF (Syngo X-workplace; Siemens). Repeat CBCT scan as required for tool in lesion.		
		CBCT scan + UTB + r-EBUS + VBN	VBN navigation added to previously mentioned technique			
		r-EBUS	Flexible bronchoscope, r-EBUS (± guide sheath)	Nil		
		r-EBUS + VBN	VBN navigation added to previously mentioned technique			
Kawakita et al^36^	Pr	VBN + UTB + r-EBUS + CBCT scan	VBN navigation, ultrathin bronchoscope (BF-MP290F; Olympus), r-EBUS (UM-S20-17S; Olympus) fluoroscopy	CBCT scan (Artis Zeego; Siemens) for tool in lesion.	Forceps, brushings, washings	Nil
Katsis et al^37^	Pr	ENB + CBCT scan + r-EBUS	ENB navigation (SuperDimension iLogic 7.2 ENB platform; Medtronic); r-EBUS probe inserted; digital tomosynthesis (Portable GE9900 Fluoroscopy C-arm; GE Healthcare) used to mark the true nodule location; ENB realigned; r-EBUS	CBCT (Philips Allura FD20 Biplane System; Phillips)-AF (Lung Suite Software; Philips) to confirm tool in lesion.	TBNA (Arcpoint 21G; Medtronic), forceps, washing	Nil
Kheir et al^38^	Re	ENB + CBCT scan + r-EBUS	ENB (SuperDimension iLogic 7.0; Medtronic), flexible bronchoscope (BF-1T180; Olympus), r-EBUS used to identify lesion	CBCT (Artis Zeego; Siemens)-AF (Syngo iGuide; Siemens) to confirm tool in lesion.	TBNA, brushings, forceps, BAL	ROSE
		ENB + r-EBUS	ENB (SuperDimension iLogic 7.0; Medtronic), flexible bronchoscope (BF-1T180; Olympus), r-EBUS used to identify lesion	Nil		
Podder et al^39^	Pr	ENB + CBCT scan + r-EBUS	ENB (SuperDimension version 7.1; Medtronic), flexible bronchoscope (BF-1TH190; Olympus America), r-EBUS to identify the lesion	CBCT scan (Aris Q Bi-Plane; Siemens) to confirm tool in lesion.	TBNA (Arcpoint 21G; Medtronic)	Nil
Sobieszczyk et al^40^	Re	ENB + CBCT scan + r-EBUS	ENB (SuperDimension navigation system 6.0; Medtronic), flexible bronchoscope, r-EBUS	CBCT scan for tool in lesion.	Transbronchial access tool (GenCut core biopsy system; Medtronic), TBNA, forceps, brush	Nil
Verhoeven et al^41^	Pr	Primary CBCT group	CBCT-AF, flexible bronchoscopy (EB19-J10; Pentax Medical), r-EBUS (UM-S20-17S; Olympus)	CBCT (Phillips Allura Clarity FD20 scanner; Philips Azurion or Artis Zeego; Siemens)-AF to confirm lesion access.	TBNA, forceps, cryobiopsy, BAL, brushing	ROSE
		Primary ENB group	ENB (SuperDimension 7.0; Medtronic), flexible bronchoscopy, r-EBUS	If ENB-/r-EBUS-confirmed lesion access confirmatory, CBCT (Artis Zeego; Siemens)-AF. If no confirmation, then CBCT-AF used to guide subsequent biopsy.		
Cumbo-Nacheli et al^42^	Re	RAB + CBCT scan + r-EBUS	RAB (Monarch; Auris), flexible bronchoscope, r-EBUS	CBCT scan to confirm tool in lesion.	TBNA (Periview; Olympus), forceps	Nil
Styrvoky et al^43^	Re	SSRAB + CBCT scan + r-EBUS	SSRAB (Ion Endoluminal Robotic Bronchoscopy System; Intuitive Surgical); Ion PlanPoint software incorporated into preoperative CT scan (Intuitive Surgical); flexible bronchoscope; r-EBUS	CBCT (Philips Allura Clarity platform; Philips)-AF (XperGuide Software; Phillips).	TBNA (19-, 21-, 23-gauge Flexision needles; Intuitive Surgical), forceps, brush	ROSE
Reisenauer et al^44^	Pr	SSRAB + CBCT scan + r-EBUS	SSRAB (Ion Endoluminal Robotic Bronchoscopy System; Intuitive Surgical); Ion PlanPoint software incorporated into preopertive CT scan (Intuitive Surgical); flexible bronchoscope; r-EBUS.	CIOS 3D Spin Mobile (Siemens) to visualize tool in lesion.	N/A	ROSE

AF = augmented fluoroscopy; CBCT = cone beam CT; ENB = electromagnetic navigation bronchoscopy; m-CBCT = mobile cone beam CT; Pr = prospective; r-EBUS = radial endobronchial ultrasound; RAB = robotic-assisted bronchoscopy; Re = retrospective; ROSE = rapid onsite cytologic examination; SSRAB = shape sensing robotic-assisted bronchoscopy; TBNA = transbronchial needle aspiration; UTB = ultrathin bronchoscope; VBN = virtual bronchoscopic navigation.

### Study Risk of Bias Assessment

Two researchers (M. V. B. and M. A.) independently used the Critical Appraisal Skills Programme tool for diagnostic studies to evaluate the methodology of the included studies. Disagreements were resolved with discussion. Results for each aspect of the assessment were reported as yes, no, or cannot tell. Certainty of results and impact of the test on the population of interest were documented in prose, and a consensus statement was agreed on. The Grading of Recommendations Assessment, Development, and Evaluation (GRADE) approach was then used by two researchers (M. V. B. and A. B.) to evaluate the quality of the outcomes. A baseline assumption about the quality of the evidence was based on study design. The assessment was then downgraded based on parameters of risk of bias, inconsistency, indirectness, imprecision of estimates, and likelihood of publication bias. Reasons to upgrade the quality of evidence were also sought including a large magnitude of effect, dose-effect gradient, and potential confounders expected to have an effect opposite to the actual effect.

### Effect Measures

The primary outcome parameter was diagnostic yield, defined as the number of nodules in which the procedure was diagnostic relative to the total number of attempted procedures. A positive diagnosis that contributed to the calculation of diagnostic yield was defined as a specific malignant or benign histologic process. A nonspecific benign pathologic diagnosis (eg, inflammation) required confirmation with repeat biopsy or surgical sampling or had consistent clinical and radiologic follow-up. Follow-up for nondiagnostic or benign results was variable among studies ranging from not specified to 2 years of radiologic follow-up. This primary outcome was analyzed for individual subgroups based on the navigation techniques used (ENB, VBN, or RAB). Primary outcome data were measured as an absolute number and percentage. The absolute number was used in the statistical analysis.

### Synthesis Methods

The diagnostic yield was pooled across studies using random effects meta-analysis, with the Freeman-Tukey double arcsine transformation applied to stabilize variance.^26^^,^^27^ Between-study heterogeneity in the diagnostic yield percentage was assessed by inspecting the forest plots and calculating *I*^2^ statistics.^28^ Subgroup analyses were performed according to the method of navigation because this was a priori expected to influence diagnostic accuracy. An additional subgroup analysis was conducted for the trials that enrolled control groups that used r-EBUS plus fluoroscopy alone, therefore facilitating a direct comparison. A sensitivity analysis was performed to test how sensitive the results were to methodologic assumptions. Further exploratory investigation into sources of heterogeneity was conducted by subgrouping and excluding studies with similar characteristics that could influence the pooled diagnostic yield and *I*^2^ value. Statistical calculations were performed using Stata statistical software version 18 (StataCorp).

## Results

### Study Selection

A total of 4,681 studies were identified based on our search criteria (Fig 1). Once duplicates were excluded, there were 3,722 articles for abstract and title assessment. After review of abstract and titles, 111 studies remained for full text review, of which 17 satisfied the inclusion criteria. Two studies were excluded because of overlapping patient populations.^25^^,^^29^ One study was excluded because the median target nodule size was 35 mm, above the 30-mm definition of a nodule.^30^ Therefore, 14 studies were available for analysis.^31^, ^32^, ^33^, ^34^, ^35^, ^36^, ^37^, ^38^, ^39^, ^40^, ^41^, ^42^, ^43^, ^44^

### Study Characteristics

Of the 14 included studies, six studies^31^^,^^36^^,^^37^^,^^39^^,^^41^^,^^44^ had a prospective design and eight studies were retrospective.^32^, ^33^, ^34^, ^35^^,^^38^^,^^40^^,^^42^^,^^43^ There were four studies that enrolled a control group.^33^, ^34^, ^35^^,^^38^ A total of 1,129 patients were included in the analysis (Table 1). This included 865 patients who underwent CBCT scan and 264 patients in control arms. A description of the navigation, role of CBCT scan, and sampling tools is included in Table 2. The CBCT equipment used included both fixed and mobile systems. A total of 882 lesions were biopsied under CBCT guidance. Mean or median nodule sizes were documented in all trials, with a maximum mean/median nodule size of 28 mm and a minimum mean/median nodule size of 12.8 mm. A total of 553 of 882 lesions (62.7%) undergoing CBCT scan were located in the upper lobes. A total of 544 of 860 lesions (63.3%) had a positive bronchus sign, and 427 of 566 (75.4%) were reported as solid lesions. Of the lesions, 142 of 318 (44.65%) were fluoroscopically visible. The mean number of CBCT scans per patient ranged from 1 to 3.5. The radiation dose and procedural duration are reported in Table 1.

### Risk of Bias in Studies

In most studies, there was a clear study question in which information was included about the population, test, and outcome of interest (Table 3). A comparison group with an appropriate reference standard was only present in five studies,^31^^,^^33^, ^34^, ^35^^,^^38^ and because of the nature of the procedure, the groups could not be anonymized. The methods for performing the test were described sufficiently in all cases. Results were expressed adequately as an absolute number with percentage diagnostic yield; however, in most cases, the certainty of these results was limited because study numbers were small and *P* values and CIs were not expressed. Additionally, there were multiple confounders across the studies that may have influenced the interpretation of diagnostic yield attributable to CBCT scan (Table 3). The challenges controlling for confounders may partly be attributable to the lack of randomized controlled trials and few prospective studies. Because of these biases, the generalizability of the data and the impact of using CBCT scan on a new population is subsequently limited.

**Table 3:** Data Quality Assessment Using the Critical Appraisal Skills Programme

Study	Was There a Clear Question for the Study to Address?	Was There a Comparison With an Appropriate Reference Standard?	Did All Patients Get the Diagnostic Test and the Reference Standard?	Could the Results of the Test Have Been Influenced by the Results of the Reference Standard?	Is the Disease Status of the Tested Population Clearly Described?	Were the Methods of Performing the Test Described in Sufficient Detail?	What Are the Results?	How Sure Are We About the Results? Consequences and Cost of Alternatives Performed?	Can the Results Be Applied to the Patients/the Population of Interest?	Can the Test Be Applied to the Patient or Population of Interest?	Were All Outcomes Important to the Individual or Population Considered?	What Would Be the Impact of Using This Test on the Patients/Population?
Casal et al^31^	Yes	Yes	Yes	Yes	Yes	Yes	Radiation dose, navigation, and diagnostic yield	Limited: pilot study with a small number of patients without a control arm. Additional confounders (ROSE and TBNA).	Cannot tell	Yes	Cannot tell	The diagnostic test is feasible and diagnostic accuracy may be similar to standard r-EBUS.
Salahuddin et al^32^	Yes	No	Yes	Yes	Yes	Yes	Diagnostic yield, sensitivity for malignancy	Fair: CIs are described. Small cohort limits accuracy. Confounders present (eg, ROSE).	Cannot tell	Yes	Cannot tell	The diagnostic test is feasible and may improve diagnostic yield to > 70%.
Yu et al^34^	Yes	Yes	No	Yes	Cannot tell	Yes	Diagnostic yield, sensitivity for malignancy	Fair: compared two propensity matched groups. *P* values are reported, CIs are not. Unclear how patients got selected into each group originally.	Cannot tell	Cannot tell	Cannot tell	Probable increase in diagnostic yield particularly for malignant lesions.
Lin et al^33^	Yes	Yes	No	Yes	No	Yes	Diagnostic yield, navigational yield, complications and radiation dose	Fair: control arm present. Data are retrospective and the original enrollment is therefore unclear. No *P* values or CIs.	Cannot tell	Cannot tell	Cannot tell	Possible improvement in diagnostic yield, particularly for small lesions.
Dibardino et al^35^	Yes	Yes	No	Yes	Cannot tell	Yes	Diagnostic yield, complications	Fair: CIs are described. Multiple confounders; however, a statistical adjustment has been made.	Cannot tell	Cannot tell	Cannot tell	CBCT scan may improve diagnostic yield compared with r-EBUS alone.
Kawakita et al^36^	Cannot tell	No	Yes	Yes	Yes	Yes	Diagnostic yield, complications	Limited: small cohort. *P* values and CIs not reported. Multiple confounders impacting on diagnostic yield (UTB, VBN).	Cannot tell	Cannot tell	Cannot tell	Unclear: possible diagnostic yield of 88% using the same technique.
Katsis et al^37^	Yes	No	Yes	Yes	Yes	Yes	Diagnostic yield, navigational yield, complications	Limited: *P* values and CIs not presented.	Cannot tell	Cannot tell	Cannot tell	Unclear: possible diagnostic yield of 72.4% using the same technique
Kheir et al^38^	Yes	Yes	No	Yes	Cannot tell	Yes	Diagnostic yield, complications	Fair: confounders (eg, ROSE). *P* values are reported. Small sample size. No CIs.	Cannot tell	Cannot tell	Cannot tell	Possible improvement in diagnostic yield using the same technique.
Podder et al^39^	Yes	No	Yes	Yes	Cannot tell	Yes	Diagnostic yield, complications	Limited: small study. No *P* values or CIs.	Cannot tell	Cannot tell	Cannot tell	Possible diagnostic yield of 76% if using ENB and CBCT scan.
Sobiesczyk et al^40^	Yes	No	Yes	Yes	Yes	Yes	Diagnostic yield, complications, transbronchial access tool diagnostic yield	Limited: small cohort. *P* values are presented but no CIs. Confounders to determining CBCT scan diagnostic yield (ENB, transbronchial access tools).	Cannot tell	Cannot tell	Cannot tell	Unclear: the diagnostic yield is dependent on the combination of all diagnostic tools. Focus was feasibility of transbronchial access tool.
Verhoeven et al^41^	Yes	No	Yes	Yes	No	Yes	Radiation exposure, diagnostic yield, navigational yield	Fair: large cohort. Paper described radiation and learning curves. Confounders impacted diagnostic yield (sampling tools, ROSE, and navigation technique). *P* values and CIs not reported.	Cannot tell	Cannot tell	Cannot tell	The use of CBCT scan is feasible and there are improvements related to the learning curve.
Cumbo-Nacheli et al^42^	Yes	No	Yes	Yes	Yes	Yes	Diagnostic yield, navigation success, procedure time, and complications	Limited: small cohort feasibility study. *P* values and CIs not presented.	Cannot tell	No	Cannot tell	Unclear impact of using this test given limitations of the data.
Styrvoky et al^43^	Yes	No	Yes	Yes	Yes	Yes	Diagnostic yield, sensitivity, specificity, negative and positive predictive values	Good: retrospective design but large cohort. CIs are reported.	Cannot tell	No	Cannot tell	Diagnostic accuracy likely between 86.7% and 94.8% if all resources are available locally.
Reisenauer et al^44^	Yes	No	Yes	Yes	Yes	Yes	Navigational yield, diagnostic yield, radiation dose, complications.	Limited: small cohort with diagnostic confounders including ROSE. *P* values and CIs not presented.	Cannot tell	No	Cannot tell	Possible improvement in diagnostic yield with the combination of CBCT scan and SSRAB.

CBCT = cone beam CT; ENB = electromagnetic navigation bronchoscopy; r-EBUS = radial endobronchial ultrasound; ROSE = rapid onsite cytologic examination; SSRAB = shape sensing robotic-assisted bronchoscopy; TBNA = transbronchial needle aspiration; UTB = ultrathin bronchoscope; VBN = virtual bronchoscopic navigation.

### Results of Syntheses

**Figure 2:**
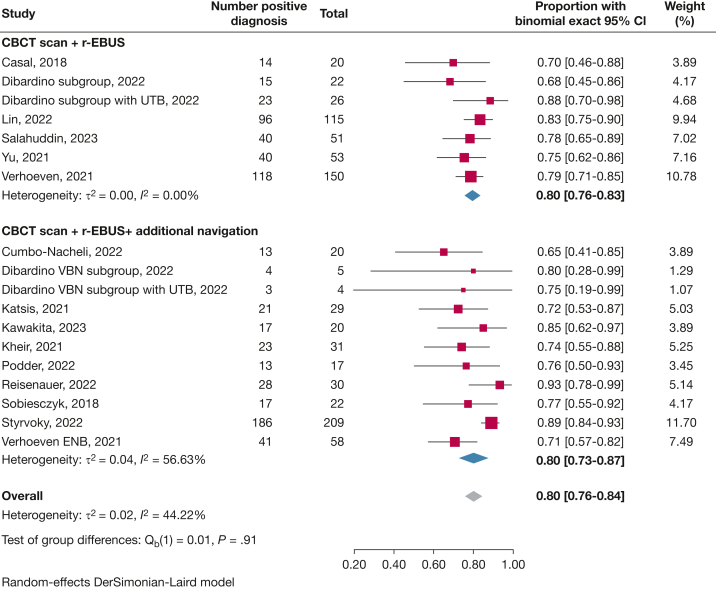
Forest plot of overall diagnostic yield. CBCT = cone beam CT; ENB = electromagnetic navigation bronchoscopy; r-EBUS = radial endobronchial ultrasound; UTB = ultrathin bronchoscopy; VBN = virtual bronchoscopic navigation.

The pooled diagnostic yield of combined CBCT scan and r-EBUS-guided biopsy for the diagnosis of PPNs was 80% (95% CI, 76%-84%) (Fig 2). Subgroup analysis was conducted based on the use of additional navigational technologies, as outlined in Table 2. For the combined use of CBCT scan and r-EBUS without additional navigational techniques, the pooled diagnostic yield was 80% (95% CI, 76%-83%). The diagnostic yield of combined CBCT scan and r-EBUS in combination with additional navigational technologies (ENB, VBN, or RAB) was also 80% (95% CI, 73%-87%).

**Figure 3:**
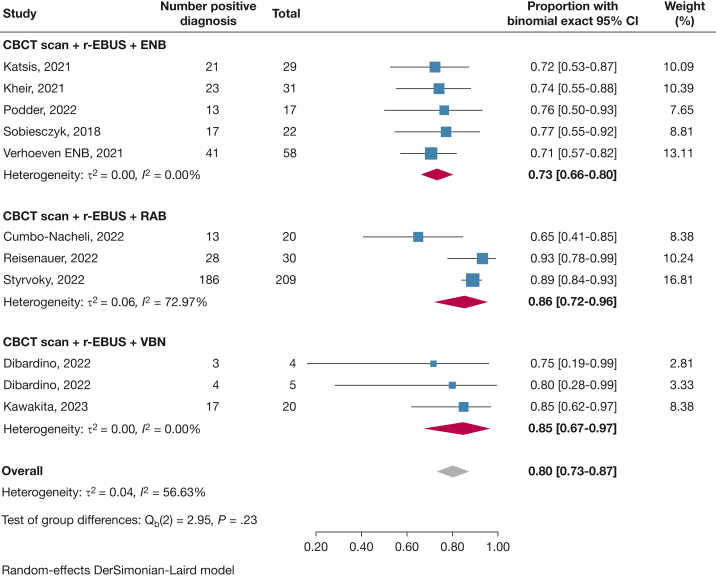
Forest plot for subgroup analysis of CBCT scan + r-EBUS + additional navigational tool. CBCT = cone beam CT; ENB = electromagnetic navigation bronchoscopy; r-EBUS = radial endobronchial ultrasound; RAB = robotic-assisted bronchoscopy; VBN = virtual bronchoscopic navigation.

Further subgroup analysis was completed to look at the breakdown of diagnostic yield by additional navigation modalities. For the combination of CBCT scan, r-EBUS, and ENB, the diagnostic yield was 73% (95% CI, 66%-80%). For CBCT scan, r-EBUS, and RAB, the diagnostic yield was 86% (95% CI, 72%-96%). The combination of CBCT scan, r-EBUS, and VBN had a diagnostic yield of 85% (95% CI, 67%-97%) (Fig 3).

The overall *I*^2^ value was 44.2%, suggesting moderate heterogeneity between trials. There was no significant heterogeneity between results for combined CBCT scan and r-EBUS alone, with an *I*^2^ value of 0.0%. Conversely, there was substantial heterogeneity in the pooled analysis of combined CBCT scan with r-EBUS plus additional navigation techniques (*I*^2^ = 56.6%).

**Supplementary Figure 1:**
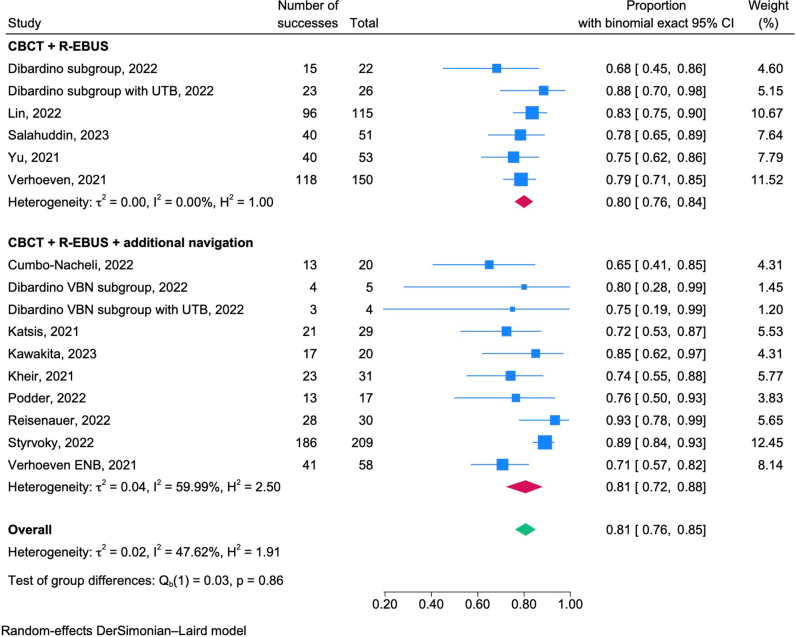
e-Figure 1: Sensitivity analysis. Studies not clearly enrolling sequential patients have been excluded

**Supplementary Figure 2:**
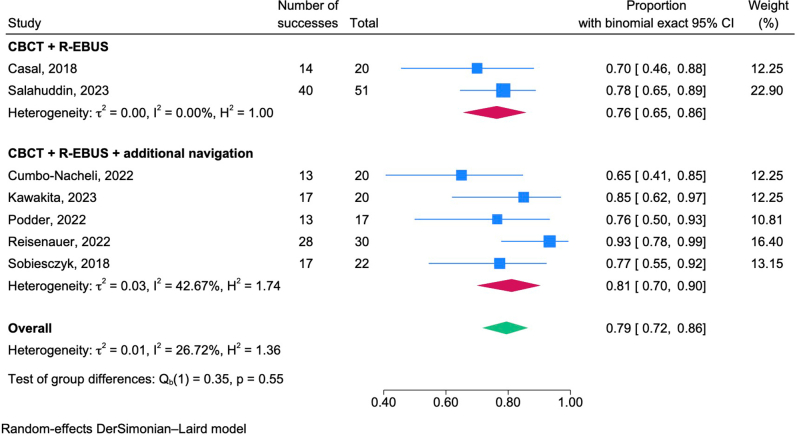
e-Figure 2: Exploratory analysis for sources of heterogeneity. Exclusion of studies utilising augmented fluoroscopy (AF)

**Supplementary Figure 3:**
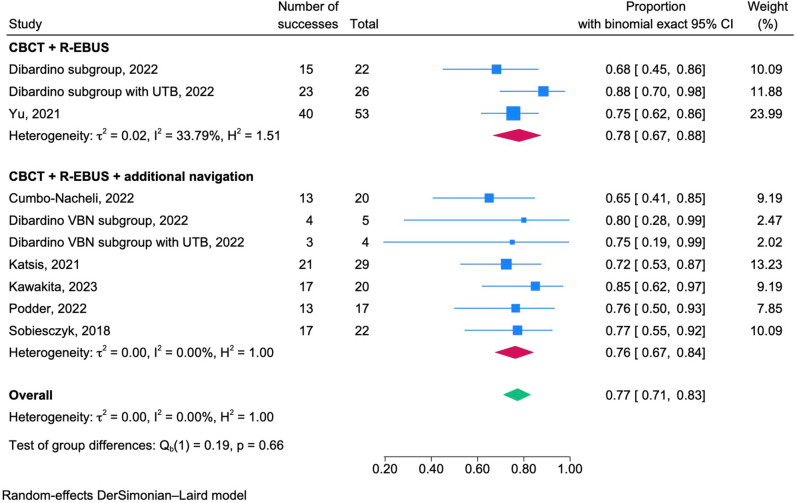
e-figure 3: Exploratory analysis for sources of heterogeneity. Exclusion of studies utilising Rapid on-site cytological examination (ROSE)

**Supplementary Figure 4:**
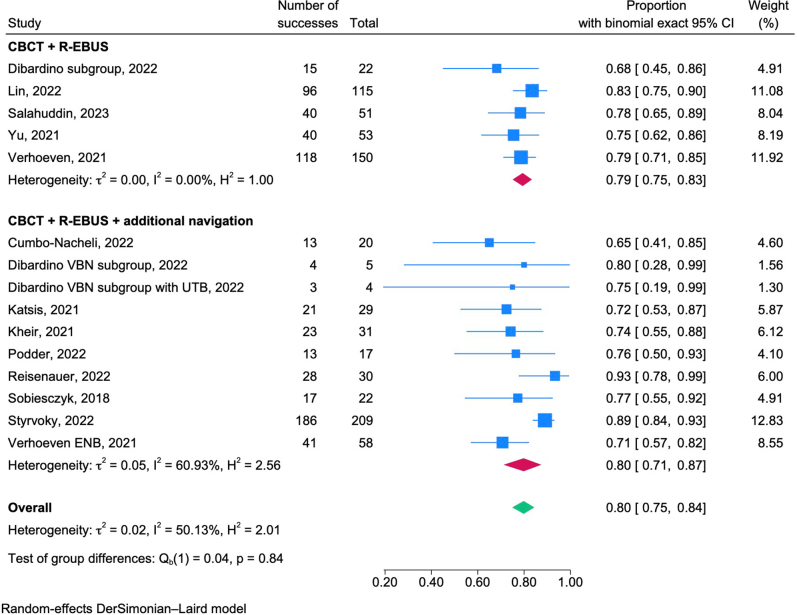
e-figure 4: Exploratory analysis for sources of heterogeneity. Exclusion of studies utilising ultrathin bronchoscopy

In sensitivity analysis, the enrollment of sequential patients was identified as a methodologic assumption. When studies that did not clearly enroll sequential patients were excluded, the pooled diagnostic yield remained at 0.81 (95% CI, 0.76-0.85) and moderate heterogeneity persisted with *I*^2^ = 47.62% (e-Fig 1). No other methodologic assumptions were identified as potentially influencing the results, and there were no obvious outliers that unduly influenced the results. Further exploratory subgroup analysis was also conducted for the impact of key study design characteristics including ROSE, AF, and UTB on overall heterogeneity and diagnostic yield. When studies using AF were excluded, the diagnostic yield remained at 0.79 (95% CI, 0.72-0.86) and the heterogeneity *I*^2^ value reduced from 44.2% to 26.72% (e-Fig 2). Similarly, the exclusion of studies using ROSE had a diagnostic yield of 0.77 (95% CI, 0.71-0.83) with a reduced *I*^2^ value of 0.00% (e-Fig 3). After the exclusion of studies using UTB, the diagnostic yield was 0.80 (95% CI, 0.75-0.84) with *I*^2^ = 50.13% (e-Fig 4). The use of ROSE and AF were therefore identified as key sources of heterogeneity in this review.

Complications rates were reported in most studies. When including only studies that reported complications, there were 13 pneumothoraxes reported out of 648 patients who underwent combined CBCT scan and r-EBUS-guided biopsy, a 2.01% pneumothorax rate. Bleeding was reported in seven instances (1.08%). Other complications were not consistently reported across the literature; hence, the incidence cannot be confidently estimated (Table 1).

Radiation doses were also reported in most studies; however, the method of reporting was heterogenous. The total radiation dose of combined fluoroscopy and CBCT scan was between 19.59 and 85.9 Gy.cm^2^, resulting in an approximate effective dose range of 3.1 to 13.8 mSv (Table 1).

A further subgroup analysis was conducted for the studies that enrolled control groups examining the diagnostic yield of r-EBUS only. Three studies were included facilitating a more direct comparison to the CBCT scan and r-EBUS group.^33^, ^34^, ^35^ The pooled diagnostic yield of r-EBUS only control patients was 58% with broad CIs (95% CI, 38%-77%). A total of 206 patients were included with high heterogeneity of *I*^2^ = 85.77%.

### Quality of Evidence

The overall quality of evidence was assessed using the GRADE assessment approach. The GRADE assessment is summarized in Table 4. All studies identified for this review are observational with intrinsic risk of bias and hence start as low-quality evidence. Most studies included in this review had appropriate eligibility criteria with sequential enrollment of patients with PPNs. There were no significant flaws to exposure or outcome, and in most cases follow-up was appropriate. The evidence was therefore not downgraded. Inconsistency was downgraded for the CBCT scan plus VBN subgroup because of wide CIs and the overall diagnostic yield and the CBCT scan plus RAB subgroup because of the wide variance in point estimates and significant heterogeneity reflected in the *I*^2^ statistic. All studies directly referred to pulmonary nodule sampling and examined diagnostic yield; hence, indirectness was not downgraded. Imprecision was not downgraded for the overall diagnostic yield and CBCT scan plus r-EBUS only subgroup because the overall number of patients included in this review is above the optimal information size (400 patients would be needed to detect a 10% improvement in diagnostic yield) and CIs do not cross the clinical decision threshold between recommending and not recommending treatment. For subgroup analysis of CBCT scan, r-EBUS, and additional navigation, numbers were small; hence, imprecision was downgraded as serious or very serious. Publication bias was not detected. Overall, the quality of evidence in the CBCT scan and r-EBUS only group was assessed as low, whereas the quality of evidence in the navigational subgroup analysis and the overall diagnostic yield assessment was downgraded to very low.

**Table 4:** Grading of Recommendations Assessment, Development, and Evaluation Approach

Outcomes	No. of Studies	Study Design	Risk of Bias	Inconsistency	Indirectness	Imprecision	Publication Bias	No. of Patients	Effect Size	Overall Quality	Importance
Overall diagnostic yield	14	Observational	Not significant^a^	Serious^b^	Not significant	Not significant	Not significant	882	0.80 (95% CI, 0.76-0.84)	Very low ⨂ OOO	Critical
Diagnostic yield of CBCT scan + r-EBUS	6^c^	Observational	Not significant^a^	Not significant	Not significant	Not significant	Not significant	437	0.8 (95% CI, 0.76-0.83)	Low ⨂⨂ OO	Critical
Diagnostic yield of CBCT scan + r-EBUS + RAB	3^d^	Observational	Not significant^a^	Serious^b^	Not significant	Serious^e^	Not significant	259	0.86 (95% CI, 0.72-0.96)	Very low ⨂ OOO	Critical
Diagnostic yield of CBCT scan + r-EBUS + VBN	2^f^	Observational	Not significant^a^	Serious^b^	Not significant	Very serious^e^	Not significant	29	0.85 (95% CI, 0.67-0.97)	Very low ⨂ OOO	Critical
Diagnostic yield of CBCT scan + r-EBUS + ENB	5^g^	Observational	Not significant^a^	Not significant	Not significant	Serious^e^	Not significant	157	0.73 (95% CI, 0.66-0.80)	Very low ⨂ OOO	Critical

CBCT = cone beam CT; ENB = electromagnetic navigation bronchoscopy; r-EBUS = radial endobronchial ultrasound; RAB = robotic-assisted bronchoscopy; VBN = virtual bronchoscopic navigation.

a Most studies report consecutive patients. Clear measurement of exposure and outcome.

b *I*^2^ value is moderately high. Wide point estimates or wide CIs. Likely unexplained heterogeneity.

c Studies include Casal et al^31^, Dibardino et al^35^, Lin et al^33^, Salahuddin et al^32^, Yu et al^34^, and Verhoeven et al^41^.

d Studies include Cumbo-Nacheli et al^42^, Reisenauer et al^44^, and Styrvoky et al^43^.

e Few patients with wide CIs. Optimal information size not met, and CIs cross the clinical decision threshold between recommending and not recommending treatment.

f Studies include Dibardino et al^35^ and Kawakita et al^36^.

g Studies include Katsis et al^37^, Kheir et al^38^, Podder et al^39^, Sobiesczyk et al^40^, and Verhoeven et al^41^.

## Discussion

The findings from this systematic review and meta-analysis suggest that combined CBCT scan with r-EBUS, with or without additional navigational technologies, appears to be a safe and effective modality for bronchoscopic biopsy of PPNs. The overall diagnostic yield of 80% (95% CI, 76%-84%) is better than the traditional combined r-EBUS and fluoroscopic guidance approach, which has a yield of approximately 70%.^6^ The combination of CBCT scan and r-EBUS with additional navigational modalities (ENB, RAB, and VBN) did not appear to significantly add to the diagnostic yield (both 80% diagnostic yields); however, the number of patients and trials corresponding to each additional navigation technique was low and CIs were wide. Within the limits of this review, the use of RAB in combination with CBCT scan and r-EBUS appeared to have the most promising effect on diagnostic yield compared with ENB and VBN. Overall complication rates were low and comparable with r-EBUS combined with fluoroscopy.

The increased diagnostic yield arising from the use of CBCT scan did come at the cost of increased radiation dose. The combined effective dose range using CBCT scan was 3.1 to 13.8 mSv. By comparison, the median effective radiation dose of fluoroscopy in the literature is in the order of 0.49 ± 0.37 mSv (range, 0.16-1.3 mSv).^45^

A prior meta-analysis and systematic review suggested that the diagnostic yield of r-EBUS with fluoroscopy guidance for the sampling of PPNs was 70.6% (95% CI, 68%-73.1%), with a complication rate of 2.8%.^6^ Registry data suggest that the diagnostic yield may be lower.^46^ Subgroup analysis of studies using r-EBUS alone in this review had a pooled diagnostic yield of 58% (95% CI, 38%-77%), lower than that described in the literature. However, the number of studies is small and CIs are wide and, although there is a signal present to suggest CBCT scan significantly adds to the diagnostic yield of pulmonary nodules compared with r-EBUS with fluoroscopy guidance alone, it must be interpreted with caution. The landscape of navigational bronchoscopy has been recently meta-analyzed and included some overlapping studies with our review. Reportedly, the overall diagnostic yield of navigational bronchoscopy was 70.9% (95% CI, 68.4%-73.25%) and the pneumothorax rate was 2.5%.^47^ The diagnostic yield for ENB-guided bronchoscopy was 70.3% (95% CI, 66%-74.2%), the yield of virtual bronchoscopy was 69.4% (95% CI, 65.3%-73.2%), and for RAB it was 76.5% (95% CI, 68.4%-82.9%). The diagnostic yield of CBCT scan was reported to be 78.2% (95% CI, 70.4%-84.4%), and when CBCT scan was combined with other multimodal navigation it was 70.9% (95% CI, 68.4%-73.2%). The results of this review are consistent with these prior meta-analyses.^10^ To our knowledge, there is no preexisting English-language literature analyzing navigational bronchoscopy exclusively in combination with r-EBUS.

Alternative methods of nodule sampling have also been investigated in recent years. The combination of r-EBUS-guided transbronchial cryobiopsy of PPNs has been reported to have a yield of 77% (95% CI, 71%-84%) within a small, recently published meta-analysis.^17^ For this procedure, there is a 29.3% minor complication rate in the form of mild to moderate bleeding and a major complication rate of 1.8%.

A limitation of this review is the significant methodologic variation across the trials regarding equipment and procedural techniques. First, differences exist in the fundamental use of the CBCT device. Seven trials had access to AF software^33^, ^34^, ^35^^,^^37^^,^^38^^,^^41^^,^^43^ Often the CBCT scan with AF cases would undergo a CBCT scan prior to the procedure, not necessarily to confirm tool in lesion, but instead relying on the combination of r-EBUS image with the overlapping AF image. Second, although all trials used flexible bronchoscopy, some trials had access to UTB, which may have influenced diagnostic yield.^31^^,^^35^^,^^36^ Analysis for sources of heterogeneity with exclusion of studies using overlapping AF and UTB minimally altered the calculation of pooled diagnostic yield. The use of AF is a likely contributor to heterogeneity in this review because analysis excluding AF lowered the *I*^2^ value (e-Fig 2). Conversely, exclusion of studies using UTB did not reduce the heterogeneity (e-Fig 4).

The nodule sampling technique also varied between the trials. Transbronchial needle aspiration with the Periview (Olympus), ArcPoint (Medtronic), or Flexision (Intuitive Surgical) needles were common sampling methods.^32^^,^^37^^,^^39^^,^^42^^,^^43^ In one trial, the GenCut core biopsy transbronchial access tool (Medtronic) was used in seven patients as part of a pilot study.^40^ Transbronchial cryobiopsy was also used to enhance diagnostic yield in one trial.^41^ A small number of trials used standard forceps and brushings alone. Variation in biopsy tool has been demonstrated to impact on the diagnostic yield and may have contributed to the outcomes in this review.^29^^,^^48^ Further subgroup meta-analysis and heterogeneity assessment for sampling technique could also not be performed because of significant interstudy variability of use.

The use of ROSE was also variable between trials and contributes heterogeneity to the outcome. Seven trials had ROSE available during the procedure.^31^, ^32^, ^33^^,^^38^^,^^41^^,^^43^^,^^44^ Exploratory exclusion of the studies that used ROSE did not impact the pooled diagnostic yield but did lower the heterogeneity *I*^2^ value (e-Fig 3). Ultimately, the impact of ROSE on diagnostic yield in bronchoscopy remains uncertain; however, there is some supporting evidence for an enhanced yield.^19^

Nodule characteristics commonly reported to enhance diagnostic yield in the literature are a positive bronchus sign and nodule size.^8^^,^^49^ The variance in mean nodule size was significant across the studies between 13 mm^41^ and 26 mm.^32^ Additional differences in the numbers of solid lesions, nodule location, and presence of a positive bronchus sign (range, 17%-95%) were also likely to have influenced overall diagnostic yields. The impact of these could not be established because of variability and inconsistency of reporting across studies.

There are also likely a number of unaccounted for sources of heterogeneity. Differences in anesthetic technique, such as the degree of positive end-expiratory pressure and the use of recruitment maneuvers which are known to impact on the degree of atelectasis and interpretation of endobronchial ultrasound images, may impact yield in different centers. Additionally, acknowledging the complexity of navigational bronchoscopy, the learning curve, and differences in proceduralist experience and training are likely to contribute to heterogeneity of outcomes.

Finally, an inherent limitation in these types of studies is the variable definition of diagnostic yield. Biopsies that are nondiagnostic or benign require repeat biopsy and/or appropriate radiologic follow-up. Inadequate follow-up or further investigation may lead to an underdiagnosis and underrepresentation of malignancy in the analysis. Follow-up periods in the studies included in this review varied from not specified to 2 years of radiologic follow-up. There was also no protocolized requirement for confirmatory rebiopsy.

A number of limitations exist in the use of CBCT scan in the clinical setting. The use of CBCT scan requires access to a hybrid operating theater or access to a mobile device. There are significant cost and infrastructure requirements; hence, availability is likely reserved for tertiary center care. CBCT scan also has the increased risk of radiation exposure to patients and staff.^41^ In the workup of malignancy, the radiation dose to a patient is often acceptable. Caution must be applied for the risk of repetitive exposure to procedural staff. The availability of protective equipment (eg, bronchoscope holders, acrylic shields) and adequate anesthetic support are required to facilitate radiation safety. Additionally, tool-in-lesion does not necessarily correlate with diagnostic sampling and false negatives occur. Despite the advances in bronchoscopic navigation, diagnostic accuracy remains considerably lower than navigation success. Traditional biopsy tools (forceps, brushings, and transbronchial needle aspiration) sample in the forward direction risking inadequate lateral biopsy. Multimodal tissue sampling can offset this; however, improving tissue acquisition methodology is likely to be crucial to enhancing diagnostic bronchoscopy success in the long-term.

Despite the limitations, the clinical implications of this review would suggest that combined CBCT scan and r-EBUS-guided biopsy for the sampling of PPNs is safe, with a diagnostic yield that may be on average higher than traditional approaches using fluoroscopy guidance. The quality of this recommendation is low to very low as demonstrated by the Critical Appraisal Skills Programme and GRADE assessments, and further prospective and randomized trials are required before a definitive recommendation can be made. Barriers to implementation include the limited availability of the technology and the learning curve of advanced navigational techniques requiring significant education and training. The increase in diagnostic yield as demonstrated in this review must also be weighed against the increased resource requirements of time, general anesthesia, and trained radiography staff, whereas standard r-EBUS with fluoroscopy can be conducted with IV sedation. Hence, the exact role of CBCT scan needs to be refined to establish the patients that will benefit most from the intervention. Some studies included in this review demonstrated an impressive diagnostic yield in small nodules.^41^ Certain lesion characteristics also require meticulous positioning such as pleurally based nodules, those with no bronchus sign, or lesions in the lower lobes where motion artifact and atelectasis can impair r-EBUS imaging providing false positives and multiple bronchi are frequently parallel. Further studies are required to identify which anatomic and lesional characteristics are best suited to the use of CBCT cross-sectional imaging. Finally, as the field of interventional pulmonology advances toward directed endobronchial therapies, the use of cross-sectional imaging is likely to be increasingly important to ensure the safe implementation of these therapies.

## Interpretation

Combined CBCT scan and r-EBUS-guided biopsy for the sampling of PPNs appears to be safe and effective. The pooled diagnostic accuracy of this combined technique is 80% (95% CI, 76%-84%), with a pneumothorax rate of 2.01% and bleeding rate of 1.08%. The diagnostic yield is higher than the pooled yield of r-EBUS plus fluoroscopy alone in this review and 10% higher than that reported in the literature. Moderate heterogeneity exists between studies, and differences in methodology between studies may impact the generalizability of this review. This is the first systematic review to investigate the combination of r-EBUS and CBCT scan in the evaluation of PPNs. With the growing evidence and implementation of lung cancer screening, further research is required to evaluate the role of modern, advanced, 3-D imaging and navigation techniques that aim to improve diagnostic yield of PPNs while minimizing complications.

## Funding/Support

The authors have reported to *CHEST Pulmonary* that no funding was received for this study.

## Financial/Nonfinancial Disclosures

None declared.

## Supplementary Data

e-Online Data
