# Supplementary material for: The Diagnostic Yield of Cone Beam CT Combined With Radial-Endobronchial Ultrasound for the Diagnosis of Peripheral Pulmonary Nodules: Systematic Review and Meta-Analysis
Source: CHEST Pulm. 2024 Jan 24;2(2):100037. doi: 10.1016/j.chpulm.2024.100037 (PMC13417805; doi:10.1016/j.chpulm.2024.100037)
Supplement: e-Online Data [file mmc1.docx]

**e-table 1**

PubMed logic grid

| Cone beam | Pulmonary nodule | Radial EBUS |
| --- | --- | --- |
| “Cone-Beam Computed Tomography”[mh:exp] OR  Cone Beam[tiab] OR  O’arm[tiab] OR Oarm[tiab] OR Computed tomography[tiab] | “Solitary Pulmonary Nodule”[mh] OR  “lung neoplasms”[mh] OR  Peripheral pulmonary lesion*[tiab] OR  Peripheral pulmonary nodule*[tiab] OR  Pulmonary coin lesion*[tiab] OR  PPL[tiab] OR  SPN[tiab] OR  Lung nodule*[tiab] OR  Pulmonary nodule*[tiab] OR  Lung neoplas*[tiab] OR Lung tumor*[tiab] OR Lung Tumour*[tiab] OR Lung cancer*[tiab] | “Bronchoscopy”[mh] OR  “Endosonography”[mh]  OR Endosonography[tiab] OR Bronchoscop*[tiab] OR  Endobronchial Ultraso*[tiab] OR Radial EBUS[tiab] OR EBUS[tiab] OR  Radial Endobronchial Ultrasound[tiab] OR  Guide sheath[tiab] OR  Radial Probe[tiab] OR  Virtual Bronchoscopic navigation[tiab] OR  VBN[tiab] OR  EMN[tiab] OR ENB[tiab] OR  Electromagnetic navigation[tiab] OR Robotic-Assisted Bronchoscopy[tiab] OR Augmented fluoroscopy |

Embase Logic Grid

| Cone beam | Pulmonary nodule | Radial EBUS |
| --- | --- | --- |
| Exp Cone Beam Computed Tomography OR  Cone Beam.ti,ab OR O?arm.ti,ab OR Computed Tomography.ti,ab | Exp lung nodule OR Exp lung tumor OR Solitary pulmonary nodule.ti,ab OR  lung neoplas*.ti,ab OR  Peripheral pulmonary lesion*.ti,ab OR  Peripheral pulmonary nodule*.ti,ab OR  Pulmonary coin lesion*.ti,ab OR  PPL.ti,ab OR  SPN.ti,ab OR  Lung nodule*.ti,ab OR  Pulmonary nodule*.ti,ab  OR Lung Tumo?r*.ti,ab OR Lung cancer*.ti,ab | Exp Endobronchial Ultrasonography OR Exp Bronchoscopy OR Bronchoscop*.ti,ab OR endobronchial ultraso*.ti,ab OR Radial EBUS.ti,ab OR EBUS.ti,ab OR Radial Endobronchial ultrasound.ti,ab OR guide sheath.ti,ab OR Radial Probe.ti,ab OR  Virtual Bronchoscopic navigation.ti,ab OR  VBN.ti,ab OR  EMN.ti,ab OR ENB.ti,ab OR  Electromagnetic navigation.ti,ab OR Robotic-Assisted Bronchoscopy.ti,ab OR Augmented Fluoroscopy.ti,ab |

Cochrane Logic Grid

| Cone beam | Pulmonary nodule | Radial EBUS |
| --- | --- | --- |
| [mh “Cone-Beam Computed Tomography”] OR  Cone Beam:ti,ab,kw OR  O’arm:ti,ab,kw OR Oarm:ti,ab,kw OR Computed tomography:ti,ab,kw | [mh “Solitary Pulmonary Nodule”] OR  [mh “lung neoplasms”] OR  Peripheral pulmonary lesion*:ti,ab,kw OR  Peripheral pulmonary nodule*:ti,ab,kw OR  Pulmonary coin lesion*:ti,ab,kw OR  PPL:ti,ab,kw OR  SPN:ti,ab,kw OR  Lung nodule*:ti,ab,kw OR  Pulmonary nodule*:ti,ab,kw OR  Lung neoplas*:ti,ab,kw OR Lung tumor*:ti,ab,kw OR Lung Tumour*:ti,ab,kw OR Lung cancer*:ti,ab,kw | [mh “Bronchoscopy”] OR  [mh “Endosonography”]  OR Endosonography:ti,ab,kw OR Bronchoscop*:ti,ab,kw OR  Endobronchial Ultraso*:ti,ab,kw OR Radial EBUS:ti,ab,kw OR EBUS:ti,ab,kw OR  Radial Endobronchial Ultrasound:ti,ab,kw OR  Guide sheath:ti,ab,kw OR  Radial Probe:ti,ab,kw OR  Virtual Bronchoscopic navigation:ti,ab,kw OR  VBN:ti,ab,kw OR  EMN:ti,ab,kw OR ENB:ti,ab,kw OR  Electromagnetic navigation:ti,ab,kw OR Robotic Assisted Bronchoscopy:ti,ab,kw OR Augmented Fluoroscopy:ti,ab,kw |

e-table 1: Logic grids for PubMed, Embase and Cochrane
